# In Vitro Antimicrobial Activity of Some Medicinal Plants against Human Pathogenic Bacteria

**DOI:** 10.1155/2019/1895340

**Published:** 2019-04-02

**Authors:** Sarita Manandhar, Shisir Luitel, Raj Kumar Dahal

**Affiliations:** Tri-Chandra Multiple College, Tribhuvan University, Kathmandu, Nepal

## Abstract

The emergence and spread of antibiotic resistance, as well as the evolution of new strains of disease causing agents, are of great concern to the global health community. Effective treatment of a disease entails the development of new pharmaceuticals or some potential source of novel drugs. Commonly used medicinal plants of our community could be an excellent source of drugs to fight off this problem. This study is focused on exploring the antimicrobial properties of the plants that are commonly being used as traditional medicines. The antimicrobial potential of four different plant extracts was screened against twelve pathogenic microorganisms and two reference bacterial strains. Methanolic extracts of* Oxalis corniculata*,* Artemisia vulgaris*,* Cinnamomum tamala*, and* Ageratina adenophora *were subjected to a test of their antimicrobial properties by agar well diffusion method. The result indicated that most of the extracts exhibited antimicrobial properties. The highest potential was observed in the extract of* O. corniculata *against* Escherichia coli, Salmonella Typhi, *MDR* Salmonella Typhi, Klebsiella pneumoniae,* and* Citrobacter koseri* with zone of inhibition (ZOI) of 17 mm, 13 mm, 16 mm, 11 mm, and 12 mm, respectively.* Oxalis corniculata* also showed the highest MIC against test organisms. The methanolic extract of* Artemisia vulgaris*,* Cinnamomum tamala,* and* Ageratina adenophora *showed efficacy against* Staphylococcus aureus. Ageratina adenophora *also showed antifungal activity against* Rhizopus *spp. The experiment confirmed the efficacy of some selected plant extracts as natural antimicrobials and suggested the possibility of employing them in drugs for the treatment of infectious diseases caused by the test organisms.

## 1. Introduction

Antimicrobial agents are essentially important in reducing the global burden of infectious diseases [1]. However, emergence and dissemination of multidrug resistant (MDR) strain in pathogenic bacteria have become a significant public health threat as there are fewer, or even sometimes no, effective antimicrobial agents available for the infection caused by pathogenic bacteria [2, 3].

Thus, in the light of the evidence of the rapid global spread of resistant clinical isolates, the need to find new antimicrobial agents is of paramount importance. However, the past record of rapid, widespread emergence of resistance to newly introduced antimicrobial agents indicates that even new families of antimicrobial agents will have a short life expectancy [4, 5].

A vast number of medicinal plants have been recognized as valuable resources of natural antimicrobial compounds as an alternative that can potentially be effective in the treatment of these problematic bacterial infections [6]. According to the World Health Organization (WHO), medicinal plants would be the best source to obtain a variety of drugs [7].

Many plants have been used because of their antimicrobial traits, which are due to phytochemicals synthesized in the secondary metabolism of the plant [8, 9]. Plants are rich in a wide variety of secondary metabolites such as tannins, alkaloids, phenolic compounds, and flavonoids, which have been found* in vitro* to have antimicrobial properties [10, 11]. A number of phytotherapy manuals have mentioned various medicinal plants for treating infectious diseases as urinary tract infections, gastrointestinal disorders, respiratory disease, and cutaneous infections.

The indigenous people of Nepal have been using many plant species as traditional medicines long ago, including treatment of infectious diseases, but there has been paucity in data regarding their* in vivo *and* in vitro* efficacy [12].

Considering the vast potentiality of plants as sources for antimicrobial drugs, this study aimed to investigate* in vitro* antibacterial and antifungal activity of extracts from some selected medicinal plants from Nepal against the most common microbial pathogens including MDR bacteria.

## 2. Materials and Methods

### 2.1. Sample Collection

Four plant materials were collected on the basis of traditional medicinal history from the local market of Kathmandu.  Table 1 shows the botanical name, family, parts used, and ethnomedicinal use of plants under this study [13–15].

### 2.2. Preparation of Plant Extracts

The collected samples were first washed under running tap water and air-dried in shade at room temperature for a month. Using a home grinder, the plant parts were then ground to fine powder. The weight of the ground powder was taken and the extract from each plant was prepared by using a cold percolation method. This 60 gram of fine powder from each plant was dissolved in 160 ml of absolute methanol at room temperature for three successive days. The supernatant was filtered through Whatman filter paper while the residues were used for a second and third extraction. Each day the dissolved parts were filtered and stored in a glass bottle. After the third extraction, the filtrates were then evaporated under reduced pressure at 50°C using a rotary evaporator to yield the crude extract.

(1) Percentage  Yield%=Dry  weight  of  extractDry  weight  of  plant  material×100

The crude extract was collected in a vial for further use.

### 2.3. Microbial Culture

A total of 12 human pathogenic microbial strains were used in the study:* S. aureus*, MRSA and* E. coli*,* Salmonella Typhi* (*S. Typhi*), MDR* S. Typhi*,* P. aeruginosa*, MDR* K. pneumoniae*,* Citrobacter koseri* (*C. koseri*), mold* Rhizopus* spp,* Aspergillus niger* (*A. niger*),* Aspergillus flavus* (*A. flavus)*, yeast* Candida albicans* (*C. albicans*), and reference strains* E. coli *ATCC 25922 and* S. aureus *ATCC 25923. A series of morphological, physiological, and conventional biochemical tests were performed to identify the selected microorganisms. The fungi were identified following growth on appropriate media and morphological and microscopic characteristics [16]. Antimicrobial susceptibility test was performed for all microbial isolates by modified Kirby Bauer disc diffusion method following the Clinical and Laboratory Standards Institute (CLSI) guideline. Multidrug resistant (MDR) isolates were defined as those isolates that are resistant to three classes of antibiotics [17].

### 2.4. Antimicrobial Assay of Plant Extracts

Antimicrobial assay of extracts of different plants was performed by agar well diffusion method in Mueller Hinton Agar (MHA) plates. The test organisms were inoculated in Nutrient broth and incubated overnight at 37°C to adjust the turbidity to 0.5 McFarland standards giving a final inoculum of 1.5 × 108 CFU/ml. MHA plate was lawn cultured with standardized microbial culture broth. Plant extracts of 50 mg/ml concentration were prepared in Dimethyl Sulfoxide (DMSO). Six wells of 6 mm were bored in the inoculated media with the help of sterile cork-borer (6 mm). Each well was filled with 50 *μ*l extracts from different plants: positive control (amikacin 30 mcg and nitrofurantoin 300 mcg) for bacteria and 1 mg/ml of cyclohexylamine for fungal isolates and negative/solvent control (DMSO), respectively. It was allowed to diffuse for about 30 minutes at room temperature and incubated for 18-24 hours at 37°C. After incubation, plates were observed for the formation of a clear zone around the well which corresponds to the antimicrobial activity of tested compounds. The zone of inhibition (ZOI) was observed and measured in mm.

### 2.5. Determination of MIC and MBC of the Plant Extracts

The broth microdilution method was used to determine the MIC according to CLSI. Twofold serial dilutions of extracts were prepared directly in a microtiter plate containing Mueller Hinton broth to obtain various concentrations. The bacterial inoculum was added to give a final concentration of 5 × 10^5^ CFU/mL in each well. The positive control was used containing amikacin as a standard drug. The plate was covered with a sterile sealer and incubated for 24 h at 37°C. Resazurin was added in each well of the microtiter plate and was incubated at 37°C for 30 min. The wells containing the bacterial growth turned into pink color whereas the well without bacterial growth remained blue. The MIC was considered as the lowest concentration of the extract that completely inhibits the bacterial growth [17].

## 3. Results

### 3.1. Extraction Yields of the Plants Extract

By using the cold percolation method, the highest yield was obtained from* C. tamala *extract of 9.35% while the least yield was of* A. adenophora *(Table 2).

Evaluation of the antimicrobial activity of four different plant extracts was determined initially by the disc diffusion method against different microorganisms. These organisms were frequently encountered in infectious diseases. The study showed that all plant extracts used in the study exhibited a varying degree of antimicrobial activity against all microorganisms tested (Table 3).

It was observed that* O. corniculata* was the most effective among the four plant extracts tested. It showed a zone of inhibition (ZOI) against all gram negative bacteria tested whereas there was no activity against gram positive bacteria and fungi.* A. adenophora* was found to be effective against both gram positive and gram negative bacteria. The extract from* A. adenophora* showed ZOI against* S. aureus* as well as* S. Typhi*. It also showed antifungal activity against* Rhizopus* spp.* C. tamala* and* A. vulgaris* showed antibacterial activity against gram positive bacteria only. Antifungal activity was shown only by* A. adenophora *against* Rhizopus *spp. None of the other plant extracts showed antifungal activity (Table 3).

The effectiveness of the extracts in tested bacterial strains was determined by measuring the minimum inhibitory concentration (MIC). MIC was performed for only those organisms which showed a zone of inhibition and were sensitive to the plant extracts in the previous antimicrobial assay by agar well diffusion method. Among all plant extracts tested,* O. corniculata* was found to show strong antimicrobial activity. The MIC of* S. Typhi* was 100mg/ml and MDR* S. Typhi* was 50 mg/ml against* O. corniculata*. Similarly, other gram negative bacteria (*E. coli, K. pneumoniae*, and MDR* C. koseri*) were found to be 25 mg/ml (Table 3). Other plant extracts were effective only against* S. aureus* with MIC value of 12.5 mg/ml (Table 4).

## 4. Discussion

Antibiotic resistance is a problem that continues to challenge the healthcare sector in a large part of the world in both developing and developed countries. The emergence and spread of multidrug resistant pathogens have substantially threatened the current antibacterial therapy. This has necessitated a search for a new source of antimicrobial substances such as plants as they produce a variety of bioactive compounds of known therapeutic properties. This study has been conducted to evaluate the antimicrobial activity of different medicinal plant extracts against human pathogens including two reference strains [2, 9, 18].

Although some extracts exhibited a good antibacterial activity towards different tested bacterial isolates, many plant extracts exhibited a limited antibacterial activity against the test bacterial isolates as judged by their MIC values.

The plant extracts of* O. corniculata *showed maximum activity against 5 pathogens,* E. coli, S*.* Typhi,* MDR* S. Typhi*,* K. pneumoniae, *and* C. koseri*. The extracts also showed significant MIC value against* E*.* coli, K. pneumoniae, *and* C. koseri.* This result was similar to those of other studies that reported antibacterial activity of methanolic extract of* O. corniculata *[19]. However, contrary to our result, they also reported antibacterial activity against* S. aureus*. The difference in result could be due to the use of plant extract in less concentration (50mg/ml) compared to that used by them (250 mg/ml) [19]. Mohan and Pandey also reported that* O. corniculata* is effective against* S. aureus*. This difference in result may be due to the use of different solvent system [20]. It has been widely observed and accepted that the medicinal value of plants lies in the bioactive phytocomponents present in the plants that dissolve in different solvent systems [21]. The emergence of multidrug resistant* S. Typhi* and* K. pneumoniae *is posing the greatest threat to mankind. The significant activity shown by the extract of* O. corniculata *against them makes us infer that it could be an important alternative to fight this nightmare.

Plant extract from* C. tamala *was found to have antimicrobial activity against only one tested bacterium,* S. aureus *(ATCC 25293). Hassan Waseem et al. [22] detected the antimicrobial activity of* C. tamala* against a number of organisms. They found different degrees of antimicrobial activity against all tested gram positive and gram negative bacteria contrary to our result where only* S. aureus* was found to be effective. Phytochemicals flavonoids, terpenoids, tannins, and alkaloids present in extracts of* C. tamala* show antihelminthic, antidiarrhoeal, and antimicrobial activities. MIC value shows that these plant extracts have the least antimicrobial activity.

*A. adenophora* was reported to produce structurally diverse chemicals including (mono-, sesqui-, di-, and tri-) terpenoids, phenylpropanoids, flavonoids, coumarins, sterols, and alkaloids that show antibacterial activity [23, 24]. The extract of* A. adenophora *was found to possess broad spectrum antimicrobial potential against* S. aureus, *MRSA, and* S. Typhi *as well as* Rhizopus *spp., which was in accordance with the result of Rajamani et al. [25]. The extract of* A. adenophora *showed a significant MIC value against* S*.* aureus *signifying that they could be a potential alternative to fight it [26]. The different nature of the cell wall makes gram positive bacteria more susceptible to different compounds than gram negative bacteria.

The extract of* A. vulgaris *showed activity against* S.aureus*. Some studies reported the antimicrobial property of* A. vulgaris *against* S. aureus *and* E. coli *similarly to our finding [27]. The extract of* A. vulgaris *also showed significant MIC value against* S. aureus*.

Although a certain number of extracts exhibited good antibacterial potency, in contrast to our expectation, a limited antibacterial potency of some plants suggests that there is no complete agreement between the traditional uses of medicinal plants in the crude form for the remedy of infectious diseases. Further study, however, is still warranted to explore their effectiveness in inhibiting the growth of parasites, viruses, and/or fungi. Another possibility for the limited antibacterial potency of some plants may be due to the cold percolation extraction method and the use of crude extracts.

## 5. Conclusion

In this study, antimicrobial activities of four traditional medicinal plants from Nepal were assessed by cold percolation method. The result showed potential antibacterial effects of* O. corniculata *extracts against bacterial strains tested, whereas* C. tamala, A. adenophora, *and* A. vulgaris *were effective only against* S. aureus.* The extract of* A. adenophora *also exhibited antifungal activity.

Even though we showed potent* in vitro *activity of a few traditional plant extracts for certain bacteria, it may not be translated* in vivo. *Instead of cold percolation method, soxhlet extraction, subfraction, semipure compound, or a pure compound isolated from these plants might exhibit better antibacterial activity. Further investigations are necessary to evaluate antimycobacterial, antiviral, and antiparasitic activity. Moreover, other parts of the plants need to be studied to evaluate the studied plant extracts as a potential antimicrobial agent.

## Figures and Tables

**Table 1:** Medicinal plants tested for their antibacterial activity in the study.

Scientific name	Family	Common name	Local name	Parts used	Traditional use
*Oxalis corniculata * (*O. corniculata*)	Oxalidaceae	Yellow sorrel	Chari amilo	Leaves	digestion, chronic dysentery, diarrhea, headaches, intoxication, fever, inflammations, jaundice, pain, scurvy, antihelminthic, analgesic, astringent, diuretic
*Cinnamomum tamala *(*C. tamala*)	Lauraceae	Bay leaf	Tejpat	Leaves	Diabetes, Digestion, Cardiovascular Benefits, Cold and Infection, Pain, Anti-cancer, Menstrual Problems
*Ageratina adenophora *(*A. adenophora*)	Compositae	Cotton weed, Eupatory	Banmara	Leaves	Cuts, wounds, boils, antiseptic
*Artemesia vulgaris * (*A. vulgaris*)	Compositae	Mugwort	Titepati	Aerial parts	Antiseptic, diarrhea, dysmenorrhea, asthma, antihelminthic, stomach ulcer, anorexia, heartburn, hyperacidity, spasm of digestive organs, epilepsy

**Table 2:** Yield percentage of methanolic extract of plants used in the study.

Plants	Solvent used	Extract yield (%)
*O. corniculata*	Methanol	4.14
*C. tamala*	Methanol	9.35
*A. adenophora*	Methanol	2.38
*A. vulgaris*	Methanol	5.57

**Table 3:** Diameter of zones of inhibition (mm) of plant extracts against microorganisms at 50mg/ml concentration.

Test organism	Plant extract			
	*O. corniculata*	*C. tamala*	*A. adenophora*	*A. vulgaris*
*E.coli*	17	-	-	-
*S. aureus*	-	10	10	10
MRSA	-	-	12	10
*S. Typhi*	13	-	13	-
MDR *S. Typhi*	16	-	-	-
*P. aeruginosa*	-	-	-	-
*K. pneumoniae*	11	-	-	-
MDR *C. koseri*	12	-	-	-
*Rhizopus* spp	-	-	11	-
*A. niger*	-	-	-	-
*A. flavus*	-	-	-	-
*C. albicans*	-	-	-	-
*E. coli *ATCC 25922	15	-	-	-
*S. aureus *ATCC 25923	-	10	10	10

(-): no antimicrobial activity.

**Table 4:** MIC value of plant extracts against microorganisms (mg/mL).

Test organism	Plant extract			
	*O. corniculata*	*C. tamala*	*A. adenophora*	*A. vulgaris*
*E. coli*	25	-	-	-
*S. aureus*	-	-	-	-
MRSA	-	-	12.5	12.5
*S. Typhi*	100	-	-	-
MDR *S. Typhi*	50	-	-	-
*K. pneumoniae*	25	-	-	-
MDR *C. koseri*	25	-	-	-
*E. coli *ATCC 25922	20	-	-	-
*S. aureus *ATCC 25923	-	12.5	25	25

(-): no antimicrobial activity.

## Data Availability

No data were used to support this study.

## References

[1] Bhatia R., Narain J. P. (2010). The growing challenge of antimicrobial resistance in the South-East Asia Region - are we losing the battle?. *Indian Journal of Medical Research*.

[2] Boucher H. W., Talbot G. H., Bradley J. S. (2009). Bad bugs, no drugs: no ESKAPE! An update from the Infectious Diseases Society of America. *Clinical Infectious Diseases*.

[3] Giamarellou H. (2010). Multidrug-resistant Gram-negative bacteria: how to treat and for how long. *International Journal of Antimicrobial Agents*.

[4] Coates A., Hu Y., Bax R., Page C. (2002). The future challenges facing the development of new antimicrobial drugs. *Nature Reviews Drug Discovery*.

[5] Marasini B. P., Baral P., Aryal P. (2015). Evaluation of antibacterial activity of some traditionally used medicinal plants against human pathogenic bacteria. *BioMed Research International*.

[6] Iwu M. W., Duncan A. R., Okunji C. O., Janick J. (1999). New antimicrobials of plant origin in. Perspectives on new crops and new uses. *Plant Breeding Reviews*.

[7] (2002). *World Health Organization*.

[8] Medina A. L., Lucero M. E., Holguin F. O. (2005). Composition and antimicrobial activity of *Anemopsis californica leaf oil*. *Journal of Agricultural and Food Chemistry*.

[9] Romero C. D., Chopin S. F., Buck G., Martinez E., Garcia M., Bixby L. (2005). Antibacterial properties of common herbal remedies of the southwest. *Journal of Ethnopharmacology*.

[10] Duraipandiyan V., Ayyanar M., Ignacimuthu S. (2006). Antimicrobial activity of some ethnomedicinal plants used by Paliyar tribe from Tamil Nadu, India. *BMC Complementary and Alternative Medicine*.

[11] Djeussi D. E., Noumedem J. A. K., Seukep J. A. (2013). Antibacterial activities of selected edible plants extracts against multidrug-resistant Gram-negative bacteria. *BMC Complementary and Alternative Medicine*.

[12] Kunwar R. M., Bussmann R. W. (2008). Ethnobotany in the Nepal Himalaya. *Journal of Ethnobiology and Ethnomedicine*.

[13] Singh A. G., Kumar A., Tewari D. D. (2012). An ethnobotanical survey of medicinal plants used in Terai forest of western Nepal. *Journal of Ethnobiology and Ethnomedicine*.

[14] Rajbhandari K. R. (2001). *Ethnobotany of Nepal*.

[15] Manandhar N. P. (2002). *Plants and People of Nepal*.

[16] Cheesbrough M. (2012). *District Laboratory Practice in Tropical Countries. Second edition update, Part 2*.

[17] (2015). *Performance standards for antimicrobial susceptibility testing: 25^th^ informational supplement (M100-S23)*.

[18] Talbot G. H., Bradley J., Edwards J. E., Gilbert D., Scheid M., Bartlett J. G. (2006). Bad bugs need drugs: an update on the development pipeline from the antimicrobial availability task force of the infectious diseases society of America. *Clinical Infectious Diseases*.

[19] Raghavendra M. P., Satish S., Raveesha K. A. (2006). Phytochemical analysis and antibacterial activity of Oxalis corniculata; a known medicinal plant. *myScience*.

[20] Mohan S. M., Pandey B. (2016). Antimicrobial activity of Oxalis corniculata Linn. *International Journal of Science and Research*.

[21] Cowan M. M. (1999). Plant products as antimicrobial agents. *Clinical Microbiology Reviews*.

[22] Hassan W., Zainab K. S. N., Noreen H., Riaz A., Zaman B. (2016). Antimicrobial activity of cinnamomum tamala leaves. *Journal of Nutritional Disorders & Therapy*.

[23] Yan Q. S., Yang J., Li H. M. (2006). Advances in the studies on the chemical components and bioactivity of Eupatorium adenophorum Spreng as an intruding species. *Journal of Beijing Normal University*.

[24] Li Y. M., Li Z. Y., Ye M. (2008). The chemical compositions and their bioactivities in the different parts of eupatorium adenophorum spreng. *Journal of Yunnan Agricultural University*.

[25] Kumar K. H., Shanmugavadivu M., Kumar R. R., Kuppsamy S. (2014). Antibacterial activity of leaf extracts of Ageratina adenophora L medicinal plants of Nilgiris Hill, Tamilnadu against human pathogens. *International Journal of Biosciences and Nanosciences*.

[26] Hiremath S. K., Kolume D. G., Muddapur U. M. (2011). Antimicrobial activity of Artemisia vulgaris Linn (Damanaka). *International Journal of Research in Ayurveda & Pharmacy*.

[27] Changhiz A., Alireza M., Ali R., Mehrdad P., Behbood J. (2014). Antibacterial activity of methanolic extract and essence of Sagebrush (Artemisia vulgaris) against pathogenic bacteria. *Bulletin of Environment, Pharmacology and Life Sciences*.

